# Executive Function, Visual Attention and the Cocktail Party Problem in Musicians and Non-Musicians

**DOI:** 10.1371/journal.pone.0157638

**Published:** 2016-07-06

**Authors:** Kameron K. Clayton, Jayaganesh Swaminathan, Arash Yazdanbakhsh, Jennifer Zuk, Aniruddh D. Patel, Gerald Kidd

**Affiliations:** 1 Department of Speech, Language and Hearing Sciences, Boston University, Boston, MA, United States of America; 2 Department for Psychological and Brain Sciences, Boston University, Boston, MA, United States of America; 3 Center for Computational Neuroscience and Neural Technology (CompNet), Boston University, Boston, MA, United States of America; 4 Harvard Medical School, Harvard University, Boston, MA, United States of America; 5 Department of Psychology, Tufts University, Medford, MA, United States of America; UNLV, UNITED STATES

## Abstract

The goal of this study was to investigate how cognitive factors influence performance in a multi-talker, “cocktail-party” like environment in musicians and non-musicians. This was achieved by relating performance in a spatial hearing task to cognitive processing abilities assessed using measures of executive function (EF) and visual attention in musicians and non-musicians. For the spatial hearing task, a speech target was presented simultaneously with two intelligible speech maskers that were either colocated with the target (0° azimuth) or were symmetrically separated from the target in azimuth (at ±15°). EF assessment included measures of cognitive flexibility, inhibition control and auditory working memory. Selective attention was assessed in the visual domain using a multiple object tracking task (MOT). For the MOT task, the observers were required to track target dots (n = 1,2,3,4,5) in the presence of interfering distractor dots. Musicians performed significantly better than non-musicians in the spatial hearing task. For the EF measures, musicians showed better performance on measures of auditory working memory compared to non-musicians. Furthermore, across all individuals, a significant correlation was observed between performance on the spatial hearing task and measures of auditory working memory. This result suggests that individual differences in performance in a cocktail party-like environment may depend in part on cognitive factors such as auditory working memory. Performance in the MOT task did not differ between groups. However, across all individuals, a significant correlation was found between performance in the MOT and spatial hearing tasks. A stepwise multiple regression analysis revealed that musicianship and performance on the MOT task significantly predicted performance on the spatial hearing task. Overall, these findings confirm the relationship between musicianship and cognitive factors including domain-general selective attention and working memory in solving the “cocktail party problem”.

## Introduction

Musical training is a rigorous activity that requires extensive auditory training and places high demands on working memory [1]. Expert musicians practice several hours per day for many years to hone their abilities, and are generally highly motivated to improve their musical skills [2]. Using basic auditory stimuli such as mistuned harmonic complexes, musicians have been shown to be better at concurrent sound segregation [3, 4] and pitch discrimination [5], and are less susceptible to informational masking [6] than non-musicians. Along with these behavioral findings, there are reports of physiological differences including evidence of a more robust auditory brainstem response to speech and music stimuli [7], higher gray matter volume in certain cerebral areas, and increased corpus callosum volume [8–10] in musicians as compared to non-musicians.

In the face of this mounting evidence of enhanced auditory expertise and neural differences in musicians, the hypothesis has been raised that musical training is causing improvements in general listening abilities, including speech perception in "noise" (meaning, generally "unwanted sound" ranging from Gaussian noise to competing talkers [11–13]). Support for this hypothesis may be found in studies that have reported better performance by musicians than non-musicians on common tests of speech-in-noise perception. For example, Parbery-Clark et al. [12] found a small but statistically significant performance advantage for young adult musicians over non-musicians in two clinical tests of speech understanding in Gaussian noise (overall effect size < 1dB between groups). In contrast, however, two recent studies [14, 15] using similar stimuli to those employed in Parbery-Clark et al. [12] reported no advantage in speech-in-noise perception for musicians compared to non-musicians (also see [16]).

It should be noted, though, that the studies discussed above were carried out in conditions that are not very representative of those encountered in typical "everyday listening". In more realistic communication environments, such as conversing in a crowded social setting, listeners are often required to follow a "target" speech signal in the presence of multiple competing "masker" speech signals which typically are spatially separated from the target, commonly referred to as the “cocktail party” problem [17, 18]. In a multi-talker situation, interfering speech maskers can affect the intelligibility of the target in multiple ways including: 1) energetic masking (EM) in which the maskers overlap in time and frequency with the target, limiting performance by producing competition between target and masker at the level of the auditory periphery and 2) informational masking (IM) in which the peripheral overlap of target and masker is not the primary factor governing performance. In conditions high in IM, the limitation on performance typically occurs because of high listener uncertainty, misdirected attention and confusions between target and masker sources. The effects of IM are thus the result of competition that occurs at physiological sites beyond the auditory periphery (e.g., [19, 20]; review in [21]).

Recently, Swaminathan et al. [13] reported that musicians performed significantly better than non-musicians on a task that emulated some aspects of the classical “cocktail party problem.” By manipulating the location and intelligibility of the masking speech, Swaminathan et al. were able to vary IM while keeping EM approximately constant. They found that the benefit for musicians depended critically on the amount of IM present, suggesting that cognitive factors *may* play a role in the observed differences between musicians and non-musicians. Although IM is thought to be due to non-peripheral factors including, potentially, cognitive limitations imposed by processes such as selective attention and working memory [21, 22], the possible role of cognitive factors in the musician advantage reported by Swaminathan et al. [13] has not been directly examined.

### Cognitive Factors and Executive Function

Executive functions are those processes that allow appropriate and self-regulated behavior, such as inhibition, goal-directed behavior, and working memory. Whether or not musical training leads to a boost of such cognitive processing skills has been widely debated (e.g., [12, 23–26]. For example, Zuk et al. [25] examined musicians and non-musicians matched for age, gender, IQ and socioeconomic status and found better performance in musicians on tasks measuring auditory working memory, cognitive flexibility, and verbal fluency. Other studies have found differences between musicians and non-musicians in multiple components of working memory [27, 28], cognitive flexibility [29], and verbal intelligence and inhibition [30] among other components of executive functioning [31]. In contrast, however, Boebinger et al.[15] found no differences in auditory working memory, cognitive flexibility and selective attention between musicians and non-musicians matched for age, gender, IQ and years of post-secondary education.

Cognitive factors, such as working memory and attention, have been shown to be important for speech perception in adverse listening environments [32–34] or for understanding degraded speech [35, 36]. There is some evidence that these cognitive factors may mediate differences between musicians and non-musicians in speech-in-noise tasks. For example, Parbery-Clark et al. [12] reported that musicians had a significantly higher verbal working memory and found a positive correlation between performance on their working memory task and performance on two speech-in-noise tests (the QuickSIN and HINT-F). In contrast, Boebinger et al. [15] found no significant difference between cognitive abilities of musicians and non-musicians, but found that across all participants non-verbal IQ was a significant predictor of individual speech reception thresholds in noise. However, to our knowledge no studies examining the relationship between cognitive factors and speech perception in noise have used a spatial listening task in which a target sentence co-occurred with independent intelligible speech streams coming from other locations. In an attempt to provide further insight into this issue, in the present study we measured EF and non-verbal IQ in musicians and non-musicians and related these measures to spatial hearing of speech (specifically, to spatial release from masking or SRM, defined below).

### Visual Attention

Musicians have been shown to have enhanced selective attention to auditory stimuli e.g., [37, 38]. However, whether this enhancement transfers to other domains such as vision is an open question. Here, too, there are conflicting findings reported in the literature. Some studies, e.g., [37, 38] have reported no differences in visual attention in musicians versus non-musicians while other studies, e.g., [39], have found significantly better performance in musicians compared to non-musicians. While the need for auditory attention is somewhat obvious, a musician must also attend to visual cues to communicate timing and expressive information to other musicians, to read music, and to follow a conductor (if one is present). Furthermore, in performance, attention to tactile cues (e.g., proprioception) is also important to make the precise body movements necessary for performance.

Studies comparing the performance of musicians to non-musicians in the visual domain are somewhat limited in number and scope. At a basic psychophysical level, musicians have demonstrated enhanced visuospatial choice reaction time, while showing no difference (vs non-musicians) in a simple visual reaction time paradigm [39]. Other studies have found enhancements for musicians in mental rotation [40, 41] which is thought to probe visuospatial cognition, and in spatial vision when working memory is involved [42]. There have been several reports of a musician advantage for visual working memory [27, 43, 44].

In the present study, we used a well-characterized visual attention task, multiple object tracking (MOT), in which subjects attended to cued target objects in the presence of highly confusable non-cued distractor objects as they moved randomly across a computer screen [45]. This task is roughly analogous to the auditory task designed to mimic cocktail party like listening situations in which the listeners are required to follow a speech signal from a target talker in the presence of highly confusable masker talkers. We used the MOT to investigate whether better performance observed in musicians compared to non-musicians in an auditory selective attention task is modality independent. Previous studies have shown that performance on the MOT task can be influenced by expertise effects. For example, radar operators and video game players perform substantially better on the MOT task than individuals without such expertise [46, 47] suggesting that prior visual experience can affect performance in the MOT task. It has been suggested that the origins of these differences are more likely to be cognitive rather than to (automatic) sensory differences, as inferred by the results of separate measures of visual short-term memory and attention switching [48].

In order to determine whether a domain-general enhancement in selective attention is involved in the musician advantage in the cocktail party problem, we measured visual attention in musicians and non-musicians using the MOT task and studied how that performance related to SRM. We hypothesized that individual performance on the MOT task would be correlated with SRM, since both tasks are high in attentional demands and performance on both tasks may be governed by individual differences in attentional capacity.

## Materials and Methods

### Subjects

Seventeen musicians (mean = 22.5 years; SD = 2.8 years) and 17 non-musicians (mean = 20.47 years; SD = 1.4 years) with normal hearing (defined as < = 20 dB HL pure-tone thresholds at octave frequencies from 250 to 8000 Hz) and no history of neurological disorders participated in the first part of the study (spatial hearing and executive function tasks). The age of the subjects from the two groups ranged from 18 years to 29 years, at the time of testing. Independent samples *t*-tests showed that the groups differed significantly in age [*t*(32) = 2.732, *p* = 0.01]. This difference in age between the groups was largely driven by 2 subjects from the musicians group who were 29 years old at the time of testing. Subjects who were categorized as musicians had at least 10 years of formal musical training, and most musicians practiced at least 5 hours a week. Subjects completed a musical history questionnaire that assessed age of onset and length of musical training (at the time of the study), primary instrument of expertise, and practice frequency and intensity (see Table 1). Nearly all individuals categorized as musicians were enrolled in the School of Music at Boston University. Subjects who were categorized as non-musicians had minimal (less than 3 years, on average) to no formal musical training, and did not report playing a musical instrument at the time or routinely participating in any musical activity (other than informal listening). All subjects were native speakers of American English. Of the 34 subjects, 8 (5 musicians) were participants in an earlier study [13] and their thresholds for the auditory tasks were not measured again for this study. A subset of 15 musicians and 15 non-musicians participated in the visual attention task. Prior to testing, measures of visual acuity were made to screen for any abnormalities. All participants were screened binocularly at 16 inches for Snellen acuity (obtaining 20/40 or better) Additionally, subjects were asked to report on frequency of video-game play, as video-game players have been shown to have enhanced performance on the MOT task (cf. [49]). Seven subjects (4 musicians) reported playing video games, with 5 subjects playing between 1–4 hours a week, and two subjects playing 14 hours/week.

**Table 1:** Primary instruments, training, and onset of training for the musician group.

Musician	Instrument	Years Training	Age (at time of testing)
1	Piano	15	24
2	Voice	12	22
3	Violin	14	21
4	Double Bass	17	23
5	Flute	18	29
6	Trombone	20	25
7	Cello	14	21
8	Violin	14.5	29
9	Flute	13	21
10	Clarinet	11	21
11	Cello	13	21
12	Trumpet	16	21
13	Percussion	13	20
14	Oboe	10	21
15	Viola	10	20
16	Tuba	19	23
17	Violin	16	23
**Mean**		**14.41**	**22.52**

### Ethics Statement

This study was approved by the Institutional Review Board protocol from the Boston University Human Research Protection Program. All subjects were fully informed about the goals of the study and provided written consent before their participation.

### Measures of cognitive abilities

#### Non-verbal IQ

The matrix reasoning subtest of the Wechsler Abbreviated Scale of Intelligence was used to measure non-verbal IQ [50]. Participants’ scaled scores were used for further analysis. Non-verbal IQ was measured in 16 musicians and 17 non-musicians.

#### Executive Function measures

The results of all executive function measures are given as normalized scores. The raw scores were normalized based on pre-determined age-specific Gaussian distributions provided for each test [51, 52].

1. Auditory working memory was assessed using the Digit span backwards subtest of the Wechsler Adult Intelligence System, Fourth Edition (WAIS-IV, [52]). Subjects were verbally presented with a series of digits and were asked to verbally recall them in reverse order. The digit span was increased from two to eight over sixteen trials, divided into 2-trial blocks. The task was discontinued if both trials within a given block were incorrect. Backward digit span is thought to prevent chunking strategies which can influence forward digit span performance. Forward digit span is not generally regarded as a measure of EF and therefore was not assessed [53].
2. Inhibition control and rule switching were assessed using the color-word interference subtest (condition 4 of Stroop test) of the Delis-Kaplan Executive Function System (DKEFS, [51]). In this task, for some word items the participants were required to read aloud the printed ink color of a conflicting colored word as quickly and accurately as possible (e.g., the word “blue” printed in green ink, for which the correct response is “green”). Other word items were outlined by a box, which required the participants to read the word and not name the ink color (e.g., if the word “blue” printed in green ink is inside a box, correct response is “blue”). A normalized score extracted from the time required to complete this task was compared between musicians and non-musicians.
3. Goal-directed behavior and cognitive flexibility were measured using the design fluency subtest of the DKEFS. The final, scored condition of the task required subjects to connect a series of dots switching between empty and filled dots to make as many different designs as possible within 60 seconds. Scores are derived from the total number of unique designs made.

### Spatial hearing task

The procedure for the spatial hearing task was identical to Swaminathan et al. [13]. On each trial, the target and masker were comprised of five-word sentences that were syntactically correct but not necessarily semantically meaningful. The sentences had the structure <name> <verb><number> <adjective> <object> and there were 8 possible words in each category [21]. One sentence was designated as the target and always began with the <name> call sign “Jane”, with other keywords being randomly selected from the available choices (e.g., “Jane took two new toys”). The masker sentences contained randomly selected <name> call-signs (excluding “Jane”) and keywords that differed from the target and from each other. The target and masker sentences were spoken by different adult female talkers selected at random on each trial from a set of seven available talkers.

Stimuli were delivered via Sennheiser HD 280 PRO headphones to listeners seated in a double-walled sound-attenuating chamber (Industrial Acoustics Company). Digital stimuli were generated on a PC outside the booth and then fed through separate channels of Tucker-Davis Technologies System II hardware. Target and maskers were spatialized using KEMAR head-related transfer functions recorded in a single-walled Industrial Acoustics Company sound booth (12 ft. x 14 ft. x 7.5 ft). Target speech was presented from 0° azimuth, and the maskers were presented either from the same location (colocated) or symmetrically separated in azimuth at ±15°.

In a given block, the maskers were fixed equal in level at 55 dB SPL and the level of the target was varied adaptively using a one-down one-up procedure that tracked the 50% correct point on the psychometric function (giving a threshold target-to-masker ratio, TMR). The target level was varied adaptively in 6 dB steps initially and then in 3 dB steps following the third reversal. Each block consisted of at least 25 trials and at least 9 reversals. Subjects were instructed to identify the keywords coming from the front uttered by the target talker (who always began her sentences with the word “Jane”). The possible responses were displayed orthographically on a computer screen. Subjects reported the perceived target keywords using the computer mouse to select the buttons showing the keywords on the screen. Correct answer feedback was provided during testing. Responses were counted as correct only if the listener successfully identified all four keywords. Each listener was tested for 2 spatial configurations (colocated and separated) with 6 estimates obtained for each spatial configuration totaling 12 runs which were completed in a single session (< 90 minutes). The ordering of the blocks was randomized across subjects. The first 2 blocks for each condition were considered practice runs and not included in the data analysis.

### Multiple Object Tracking

**Fig 1:**
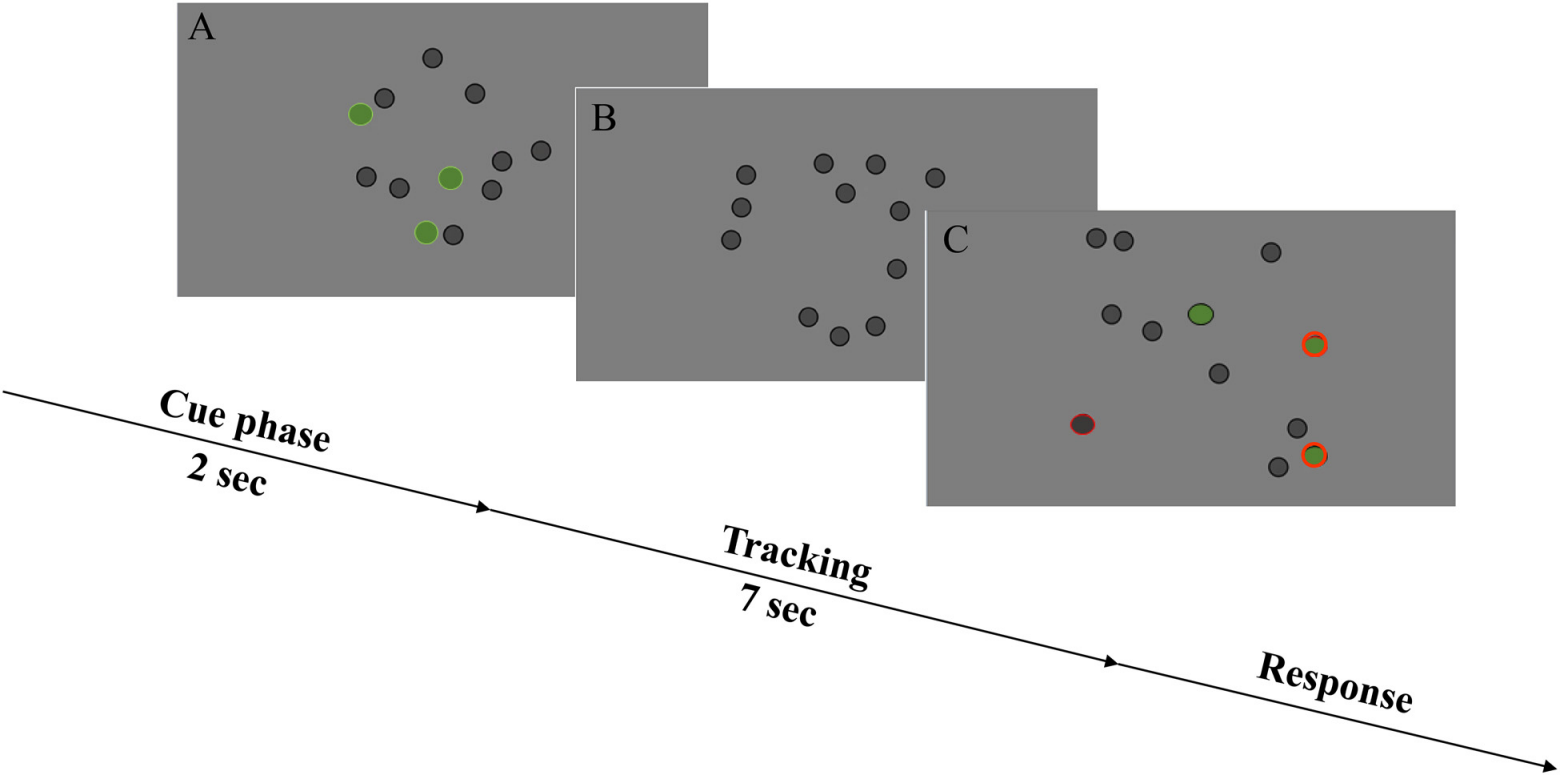
Multiple object tracking task. (A) Cue phase: Target dots, between 1 to 5, are marked in green for 2 seconds to designate them as targets for the tracking task. (B) Tracking phase: target dots turn back to grey. All of the dots, now identical, move around randomly on the display for 7 seconds. (C) Response phase: subjects report the final locations of the target dots and receive feedback on correct and incorrect choices (grey dot with red outline = incorrect selection, green dot with black outline = target dot but not selected, and green dot with red outline = target dot and selected).

Subjects tracked target dots (number of target dots or *ndots* = 1,2,3,4,5) in a field of 12 total dots presented on a screen, e.g., [45, 54]. The range of target dots tracked was selected to measure the entire performance range from easy (*ndot* = 1) to extremely difficult (*ndot* = 5), with the expectation being that robust individual differences would emerge in the intermediate conditions, i.e. *ndots* = 3, where the task was neither very easy nor overly difficult. Each trial consisted of three phases (Fig 1). In the first phase, the subject was cued to attend to a certain number of target dots, highlighted in green color. In the second phase, all dots turned grey and moved around (with a slow, smooth drifting motion) for 7 seconds. The motion of dots was randomized and speed was fixed at 7 degrees of visual angle/second. Dots bounced off the edges of the screen but not off of each other, which meant that brief occlusion was possible. In the final phase, subjects reported on the final location of the target dots using a mouse to click on the dots. Feedback was given on every trial, with the correct response displayed on the screen. Each session consisted of six blocks of 30 trials and number of dots tracked was randomized from trial to trial. The first block of 30 trials (6 runs/ndot; randomized) was used as a training block and was not scored. Performance was quantified in terms of tracking capacity as *ndots* correct/*ndots* cued. For example, if in a trial with *ndots* = 3 the observer tracked 2 out of 3 dots correctly, tracking capacity = 2/3 or .66.

Stimuli were generated in MATLAB (Mathworks, Natick, MA) with PSYCHTOOLBOX [55] and projected onto a large screen (145 cm x 84 cm projection) using a 120-Hz Optoma Projector (1280 x 720 resolution). Subjects sat in free viewing conditions at a point 135 cm from the screen and were centered at a point 42 cm from either edge of the screen.

## Results

### Spatial hearing task

**Fig 2:**
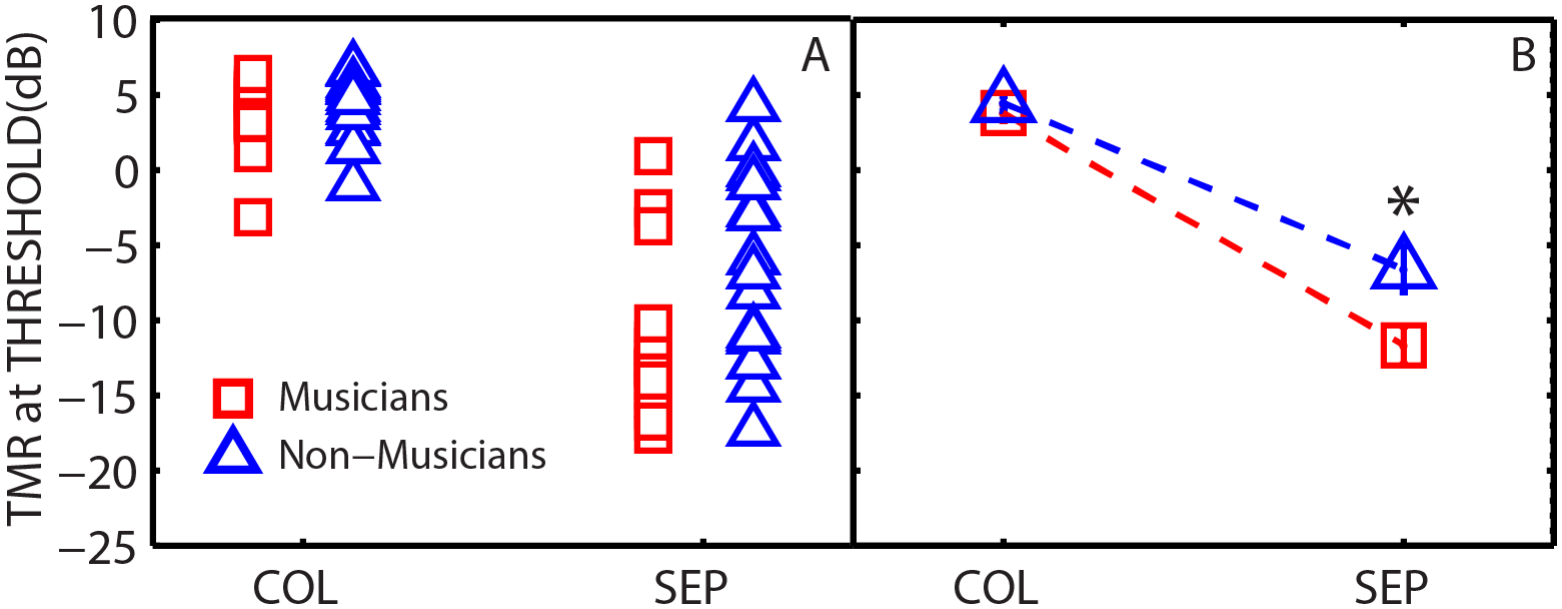
Panel A: Individual target-to-masker ratio at threshold (TMR) for musicians (red squares) and non-musicians (blue triangles) measured in colocated and separated configurations. Panel B: Group mean TMRs for conditions shown in panel A. Error bars are ±1 standard error of the mean. *Statistically significant group difference at 0.05 level (2 tailed).

Fig 2 shows target-to-masker ratios (TMRs) at threshold (calculated as the target level minus the masker level in dB) for individual subjects (panel A) and group means (panel B) for musicians and non-musicians. The TMRs are plotted for colocated and separated masker configurations. Lower TMRs correspond to better thresholds (less masking).

A two-way repeated-measures ANOVA on the thresholds revealed a significant effect of spatial configuration [*F*(1,32) = 227.5, *p* < 0.001, partial ƞ^2^ = 0.877], listener group [*F*(1,32) = 5.5, *p* = 0.025, partial ƞ^2^ = 0.147], and a significant interaction [*F*(1,32) = 6.3, *p* = 0.017, partial ƞ^2^ = 0.165]. Homogeneity of variance assumptions were confirmed using Levene’s test of equality of error variances for both colocated [*F*(1,32) = 0.001, *p* = 0.970] and separated conditions [*F*(1,32) = 2.336, *p* = 0.136]. When the speech maskers were colocated with the target, mean thresholds were similar for musicians (M) and non-musicians (NM) (M: 3.8 dB; NM: 4.4 dB). However, the musicians achieved substantially lower thresholds than non-musicians when the maskers were spatially separated from the target (M: -11.7 dB; NM: -6.6 dB). The simple subtraction of the thresholds in the two configurations indicates that musicians achieved a substantially larger SRM than non-musicians (M: 15.5 dB, NM: 11.1 dB). Independent samples two-tailed *t*-tests confirmed that the difference in SRM between musicians and non-musicians was significant [*t*(32) = 2.512, *p* = 0.017]. Among the musicians, there was no significant relationship between the separated thresholds and duration of musical training or age of onset of musical training. Overall, these results are consistent with the findings of Swaminathan et al. [13].

Large individual differences were observed in the separated thresholds, ranging over 22 dB across the two groups (from 4 to -18 dB). Amongst the non-musicians, the separated thresholds ranged from 4 dB to -17 dB. Amongst the musicians, the separated thresholds ranged from -10 dB to -18 dB for 14 of the 17 subjects with the thresholds range being higher for 3 subjects (from 1 to -4 dB). There was a weak, yet significant correlation between the age of the participants and the separated thresholds [*r*(32) = -0.349, *p* = 0.043]. There was no significant correlation between the age of the participants and the colocated thresholds or SRM.

### Cognitive tasks

Two-tailed independent sample *t*-tests showed that the participants’ with musical training had significantly better scores than non-musicians in the Backward Digit Span test, which probes auditory working memory [*t*(32) = 3.024, *p* = 0.005]. No differences in performance were observed between the groups for Matrix Reasoning (non-verbal IQ) [*t*(30) = 1.215, *p* = 0.234], Color-Word Interference (inhibition control and rule switching) [*t*(32) = 0.0, *p* = 1.0] or Design Fluency (cognitive flexibility) [*t*(32) = -0.577, *p* = 0.568] tasks. Group means for each task are presented in Table 2.

**Table 2:** Group characteristics of musicians and non-musicians for cognitive tasks. Statistically significant group differences are highlighted in bold (*p*<0.01).

Measures	Musicians norm score (mean ± SD)	Non Musicians norm score (mean± SD)
Design Fluency	11.94±2.75	12.47±2.60
Color Word Interference	12.29±2.14	12.29±1.57
**Digit Span Backwards**	**11.82±2.70**	**9.47 ±1.74**
IQ	57.13±8.57	52.94±10.79

### Multiple Object Tracking

**Fig 3:**
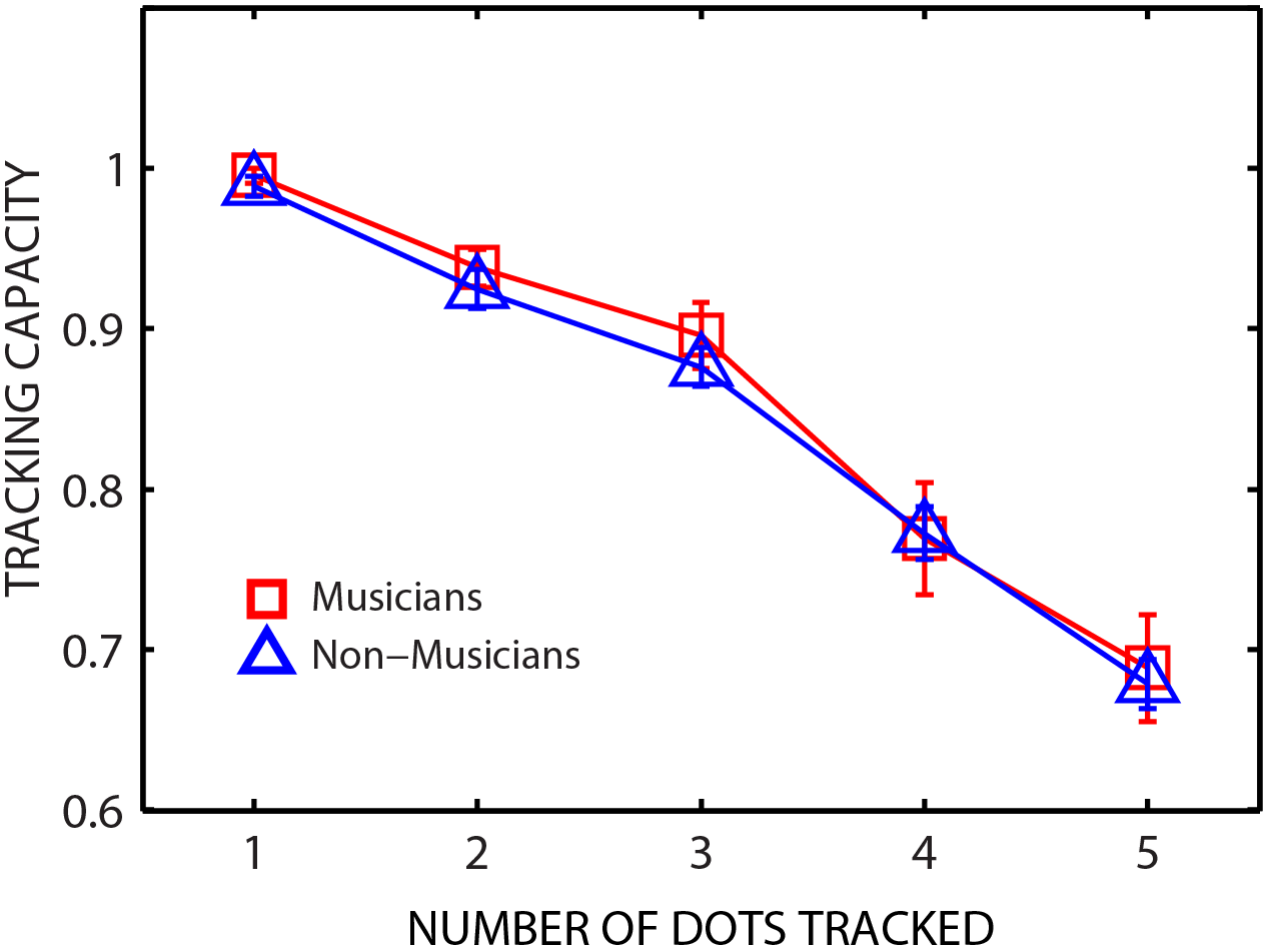
Performance of musicians and non-musicians in the multiple object tracking (MOT) task. Group mean tracking capacity data for musicians (red squares) and non-musicians (blue triangles). Error bars show ±1 standard error of the mean.

Fig 3 shows mean results for the MOT task from 15 musicians and 15 non-musicians. The MOT data was analyzed for tracking capacity, computed for each *ndot* tracked as: (*ndots* correct) / (*ndots* cued).

Across both groups, average tracking capacity was close to 1 for the easiest condition (*ndot* = 1) and decreased with increasing difficulty of the task (higher *ndots*). The tracking capacity was comparable for musicians and non-musicians for all *ndots*. A two-way repeated measures ANOVA on the number of trials correct revealed a significant effect of number of dots tracked [*F*(4,112) = 157.98.181, *p*<0.001, partial ƞ^2^ = .849] while showing no significant effect of listener group [*F*(1,28) = 0.217, *p* = 0.645, partial ƞ^2^ = .008] or interaction [*F*(4,112) = .153, *p* = .961, partial ƞ^2^ = .005].

### Relationship between Executive Function, Multiple Object Tracking, and Spatial Hearing

**Fig 4:**
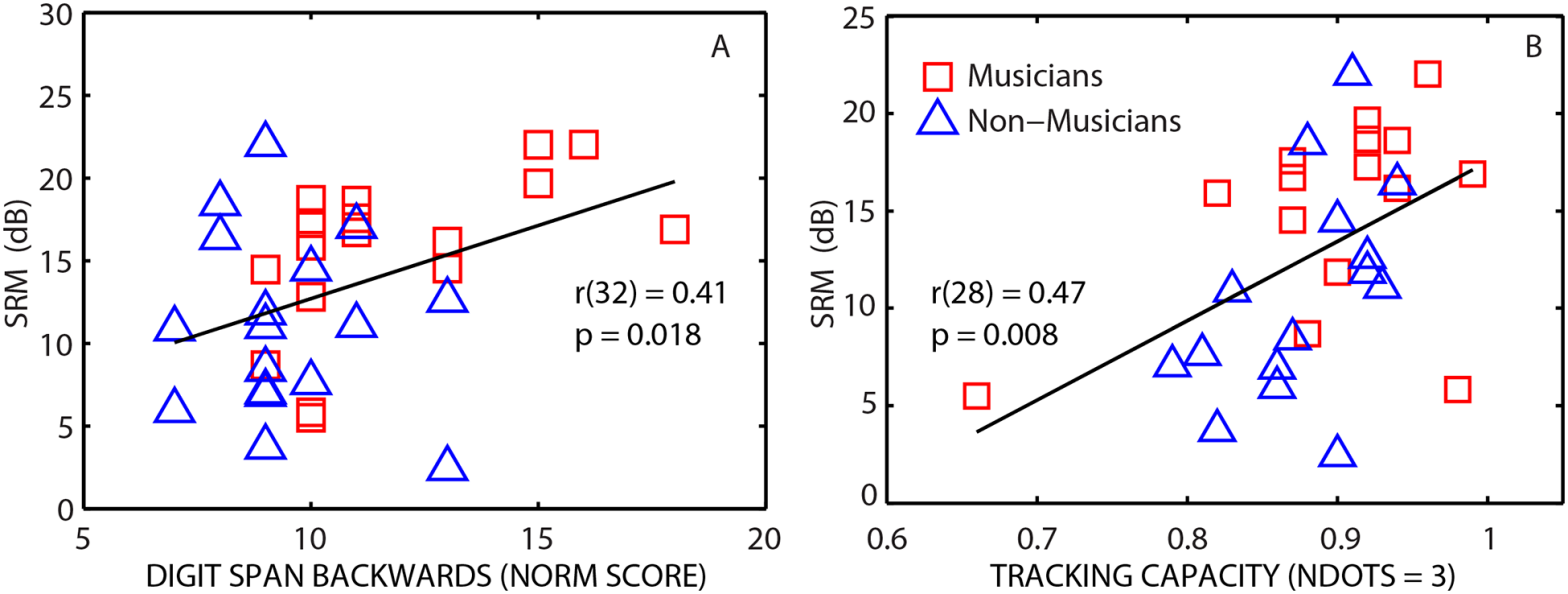
Panel A: Scatter plot shows spatial release from masking (SRM = colocated—separated thresholds) plotted against digit span backwards score for individual subjects. Panel B: Scatter plot shows SRM plotted against tracking capacity for *ndots* = 3. Solid line shows least-squares fit to the data points.

To examine the relationship between the auditory task and measures of cognitive abilities and visual attention, bivariate correlational analyses were conducted between SRM, EF and MOT measures in musicians and non-musicians. To reduce the number of correlations, only results from planned comparisons are reported. Across all EF measures, the digit span backwards (DSB) was the only test that showed differences between the musicians and non-musicians. Hence, the DSB scores were used for further analysis. In the MOT task, for *ndots* = 3, the visual attention task presented moderate difficulty compared to other *ndots* (performance near ceiling for *ndots = 1&2* and very difficult for *ndots* = 4 & 5 for both groups). Hence for the MOT task, tracking capacity for *ndots* = 3 was selected for further analysis as it was the most informative condition. SRM, digit span backwards scores (DSB), and tracking capacity for *ndots* = 3 were used as variables in the bivariate correlational analyses. Results from the Pearson correlational analyses indicated that SRM was significantly correlated with DSB task (*r* = 0.405, *p* = 0.017; Fig 4A) and MOT task (*r* = 0.471, *p* = 0.009; Fig 4B). There was also a significant correlation between the digit span backwards task and the MOT task (*r* = 0.419, *p* = 0.021). With a conservative Bonferroni correction (α = 0.017), SRM and tracking capacity were significantly correlated and SRM and DSB were just marginally correlated. No correlation was observed between SRM and non-verbal IQ measures.

To predict spatial release from masking measured from musicians and non-musicians, a stepwise multiple regression analysis was conducted with SRM as the dependent variable and listener group (LG: coded as 1 = musicians, 0 = non-musicians), DSB and tracking capacity for *ndots* = 3 (TC) as independent variables. A significant regression equation was found, [F(2,27) = 7.713, *p* = 0.002, adjusted *R*^2^ = .316]. The regression model (also see Table 3) contained only tracking capacity (*p* = .013) and listener group (*p* = .021) as significant predictors. Tracking capacity (for *ndots* = 3) accounted for almost 20% of the variance while including musicianship status accounted for an additional 12% of the variance. Collinearity diagnostic tests produced variance inflation factor values in the range of 1.022 to 1.646, indicating little redundancy among predictor variables and confirming that multiple collinearity was not a problem. Including other predictor variables such as design fluency (DF), color-word interference (CW) and matrix reasoning scores (IQ) did not yield any significant incremental explanation of the variance in SRM.

**Table 3:** Predictive model of spatial release from masking and separated thresholds based on tracking capacity (TC, for *ndots* = 3) and listener group (LG) as predictor variables.

	*Coefficients*	*t*	*Sig*	*Coefficients*	*t*	*Sig*
		SRM			Separated Thresholds	
*(Constant)*	-21.554	-1.780	0.086	34.957	2.731	**0.011**
*TC*	36.765	2.676	**0.013**	-46.753	-3.218	**0.003**
*LG*	4.364	2.449	**0.021**	-4.378	-2.324	**0.028**

Similar to predicting SRM, a stepwise multiple regression analysis was conducted to predict thresholds in the 2 masker separated configuration. A significant regression equation was found, [*F*(2,27) = 9.179, *p* = 0.001, adjusted *R*^2^ = 0.361] with tracking capacity (*p* = .003) and listener group (*p* = .028) as predictors (also see Table 3). Tracking capacity (for *ndots* = 3) accounted for almost 26% of the variance while including musicianship status accounted for an additional 10% of the variance. Overall, the results from the regression analysis show that spatial release from masking and separated thresholds were primarily predicted by tracking capacity from the MOT task and status of musical experience in the listeners.

## Discussion

The current study examined whether the benefits shown by musicians in a task emulating the classical “cocktail party problem” were related to better cognitive processing, as measured by tests of executive function and selective attention. In the spatial hearing task, we found that musicians were better able to understand target sentences masked by intelligible sentences coming from other spatial locations, but no better at understanding target sentences masked by intelligible sentences coming from the same spatial location. Thus, the overall difference in spatial release from masking (SRM) in the two groups (~ 4.4 dB) was driven almost entirely by a musician benefit in the spatially-separated condition. The colocated configuration is high in both energetic and informational masking (EM and IM), and it appears that this difficult baseline condition requires the target to be the loudest source in the mixture in order for it to be understood (i.e., TMRs > 0 dB). However, spatially separating the maskers takes the listeners out of this TMR region indicating that the listeners experienced reduced IM (e.g., [20, 56, 57]). It is in this condition that musicians achieve substantially lower thresholds than non-musicians (difference of ~ 5.1 dB). This may be attributed to their enhanced ability to suppress irrelevant background sounds, which suggests that musicians are less susceptible to IM than non-musicians, consistent with the findings of Swaminathan et al. [13].

To determine whether cognitive factors did indeed play a role in this musician advantage, we measured executive functions, non-verbal IQ, and selective attention and related these measures to performance in the spatial hearing task in musicians and non-musicians. Within our limited set of EF measures we found that a measure of auditory working memory (digit span backwards) was the only measure in which the musicians differed significantly from non-musicians. This result is in general agreement with some prior studies that have shown better auditory working memory in musicians than in non-musicians [12, 25, 27, 28]. Although differences in working memory cannot be attributed to musical training *per se*, it is plausible that musical training could enhance working memory [58–60] or that only individuals with enhanced working memory tend to be successful as musicians. It has to be noted that the digit span backwards task employed in this study may not be sensitive enough to measure and disentangle the contributions of different aspects/mechanisms associated with auditory working memory [61]. Thus there is a strong need to use and replicate the current findings with other measures of auditory working memory.

Some inconsistencies were also noted in the relationship between musical training and specific components of EF measured. For instance, we did not observe previously reported differences in cognitive flexibility [25] and inhibition [31] between musicians and non-musicians. Furthermore, Boebinger et al. [15] found no significant difference between musicians and non-musicians in a variety of EF measures including auditory working memory, inhibition and cognitive flexibility. It is plausible that the mixed findings reported between studies on EF performance in trained musicians (vs non-musicians) are likely due to various methodological limitations regarding the validity of the assessments employed and the subject inclusion criteria. Overall, the inconsistencies observed between this and other studies provide further evidence that the relationship between musical training and general cognitive abilities is complex and need further investigation (also see [61]).

Across all listeners, a marginally significant correlation was observed between auditory working memory (measured using digit span backwards test) and performance on the spatial hearing task (SRM) with higher digit span score associated with higher SRM. This result is in general agreement with the findings of Parbery-Clark et al. [12] in which a significant correlation was observed between musicians’ and non-musicians’ auditory working memory and speech in noise performance, albeit with energetic maskers (speech-spectrum noise and a four-talker babble). The results from the present study suggest that auditory working memory may also be important for performance in speech on speech masking conditions that are high in IM.

No correlation was found here between a non-verbal IQ measure and performance on the spatial hearing task. This result is in contrast to the findings of Boebinger et al. [15] in which a similar non-verbal IQ measure was found to be a significant predictor of performance on masked speech tasks in which the maskers were designed to produce varying amounts of IM. It should be noted, however, that the stimuli used in the present experiment (same-sex talkers, intelligible speech) likely presented much more difficulty in terms of cognitive confusability (and IM) than the maskers used in the Boebinger et al. study (different sex talkers, spectrally rotated speech and speech-shaped noise maskers). The findings from our study are in general agreement with a recent study by Ruggles et al. [14] in which no significant relationship was observed between IQ and masked speech thresholds with energetic maskers. Hence, the role of IQ measures and their influence on speech perception in noise is unclear and requires further exploration.

The issue of selective attention was examined by comparing musicians and non-musicians on a multiple-object tracking (MOT) task which requires attentive tracking of non-linguistic stimuli in the visual modality. We found that musicians and non-musicians did not differ significantly in performance on the selective visual attention task as measured using MOT (for all *ndots* tracked). This result is in general agreement with other studies that have reported no differences in visual attention abilities between musicians and non-musicians (e.g., [37, 38]). However, some studies have reported enhanced visual attention abilities in musicians compared to non-musicians (e.g., [62]). The origins of these discrepant findings are unknown, but may be attributed to differences in experimental procedures and/or the specific stimuli used. Across all listeners, a significant correlation was observed between performance on the visual attention task and the spatial hearing task (Fig 4B), suggesting that a domain-general attentional mechanism may mediate performance in a cocktail-party-like environment.

A significant correlation was also observed between performance on the visual attention and auditory working memory tasks. This result is interesting in light of literature on the working memory demands of the multiple object tracking task (e.g., [63]) and suggestive of visuospatial memory demands of the digit span backwards task [64]. Several studies have shown the role of visuospatial resources in the backwards digit span task, both behaviorally (e.g., [65]) and with evidence from neuroimaging (e.g., [66]). The correlation seen here between performance on the visual selective attention and auditory working memory tasks highlights the complex relationship between selective attention and working memory in perception of complex auditory and visual scenes, and the potential cognitive overlap between the two. Future work should also examine the relationship between spatial hearing and other more domain-general components of working memory (e.g., the executive, cf. [67]).

Having found several factors that were correlated with performance on the spatial hearing task (i.e. auditory working memory, visual attention, and musicianship), we conducted a stepwise multiple regression analysis to assess the contributions of each factor to explaining variability in the spatial hearing task across all subjects. In this analysis, the EF measures, IQ, performance from the MOT task, and musicianship status were used as regressor variables to predict performance on the spatial hearing task (SRM and separated thresholds). The results from this analysis showed that a two factor model including performance on the visual attention task and musicianship status best accounted for individual variability in SRM and separated thresholds.

The current study reports differences in auditory working memory in musicians and non-musicians and highlights the influence of musicianship and cognitive factors, including selective attention mechanisms that act across sensory domains in predicting performance in a masked speech recognition task with high IM. However, it should be noted that not all speech-on-speech masking conditions produce high IM and so the effectiveness of stimulus variables (e.g., spatial separation, voice differences, linguistic effects, etc.) in reducing IM varies considerably across studies (e.g., [22, 57, 68]). This means that the predictive value of the selective attention and working memory tasks found in this study likely are most revealing for masking conditions that cause significant IM. The extent to which natural listening situations (e.g., actual "cocktail parties" or similarly complex acoustic environments) consist of EM and/or IM is an intriguing but open question because, typically, such situations lack the necessary experimental controls. Thus, finding valid ways to assess the masking at play in natural listening situations is a topic of great interest for future speech intelligibility studies. While the current study does not entirely explain the extent of individual variability or group differences in complex, multi-source acoustic environments, it does provide preliminary evidence of cognitive factors that may be important for solving the “cocktail party problem”. These findings are consistent with models which view executive function as a potentially important link between music processing and other cognitive abilities such as language [69]. Although not addressed directly in this study, several studies have also shown sensory enhancements for signals in noise in the absence of goal directed attention in musicians compared to non-musicians (e.g., [70]). However, most of these studies have used steady-state noise maskers that are energetic in nature. In the context of our study, with speech-on-speech masking, it is less clear how the masker features might be selectively attenuated in lower auditory areas due to enhanced sensory representation of the target features in musicians compared to non-musicians, especially at lower (degraded) target-to-masker ratios. Nevertheless, it remains a possibility that enhanced sensory representation of acoustic features in musicians compared to non-musicians could have also contributed, at least partially, to the differences between the groups in the speech-on-speech masking task.

As with any cross-sectional study, we cannot infer from the current results that musical training *caused* improvements and led to *enhancements* in cognitive abilities and perceiving speech in noise in musicians compared to non-musicians. The issue of causality can only be addressed by longitudinal training studies with random assignment of individuals to musical training and to other forms of training (or no training), guided by specific hypotheses for how and why musical training would influence speech processing.

For example, the OPERA expanded hypothesis [60] argues that when music and speech share brain networks involved in sensory or cognitive processes, and music places higher demands on those processes than does ordinary speech communication, then neuroplastic changes to those networks caused by musical training will enhance speech processing. When this hypothesis was proposed, auditory working memory was suggested as a cognitive process 1) that engages overlapping cortical networks in music and speech processing and 2) is subject to greater demands in instrumental music than in speech processing. These greater demands were argued to arise from the need to remember extended sound patterns in working memory as part of music processing (e.g., to recognize one phrase as a variant of another), vs. in language processing, where perceived sounds can be immediately recoded into referential meanings (semantics), so that extended sound patterns need not be stored in working memory. Thus while language processing does require working memory (e.g., to link semantically and syntactically related words during sentence comprehension), its demands on this cognitive process may not be as high as that music. In principle, this could lead to the kind of auditory working memory benefits seen in musicians in the current study, with concomitant benefits to “cocktail party” listening. Whether or not a causal chain exists, however, is a topic for future work. While the current work cannot speak to issues of causality, it does motivate such work by finding associations between musical training, enhanced speech perception in multi-talker environments, and cognitive factors.

## Supporting Information

S1 File Worksheet contains all the relevant data for musicians and non-musicians. (XLSX)
